# The dark genome and pleiotropy: challenges for precision medicine

**DOI:** 10.1007/s00335-019-09813-4

**Published:** 2019-08-23

**Authors:** Steve D. M. Brown, Heena V. Lad

**Affiliations:** MRC Harwell Institute, Harwell, OX11 0RD UK

## Abstract

Surprisingly we remain ignorant of the function of the majority of genes in the human and mouse genomes. The dark genome is a major obstacle to the interpretation of the function of human genetic variation and its impact on disease. At the same time, pleiotropy, how individual variants influence multiple phenotypes, is key to understanding gene function and the role of genes and genetic networks in disease systems. Both understanding the genetics of disease and developing new therapeutic approaches and advances in precision medicine are all compromised by our limited knowledge of gene function and pleiotropic effects. Illuminating the dark genome and revealing pleiotropy across the genome requires a highly coordinated and international effort to acquire and analyse high-dimensional phenotype data from model organisms. We describe briefly how the International Mouse Phenotyping Consortium is addressing these challenges and the novel features of the pleiotropic landscape that are revealed by functional genomics programmes at genome-wide scale.

## Introduction

Numerous studies have employed GWAS and genome sequencing approaches to identify loci involved with a wide variety of human traits and diseases (Gurdasani et al. 2019). From rare diseases and Mendelian disorders to common, complex diseases, there have been a plethora of investigations to identify DNA variants, both coding and non-coding, associated with human disease. Beyond genetic approaches in the population, the candidature of specific genetic variants is aided by functional knowledge of the protein products encoded by the genes that contain variants or which reside in the vicinity of non-coding variants. Protein function as determined by studies of molecular function, cellular and tissue expression patterns, and interacting partners provide an indicator of causality, as do studies of mutations in model organisms. The eventual determination that a specific human genetic variant is causal for disease, thus establishing a genome–phenotype relationship, is a further elaboration of gene and protein function.

The challenge of the Precision Medicine initiative (Collins and Varmus 2015) is to chart the landscape of genetic variation and its impact upon disease. Establishing a comprehensive understanding of how different genetic variants within the human population impact upon developmental and physiological functions and disease states will be critical. Importantly, deciphering pleiotropy, the multiple functions of a gene and its variants within many genetic contexts (Riordan and Nadeau 2017), will be pivotal for understanding the genetic complexity, comorbidities and variability of disease states, assisting diagnosis, target discovery, therapeutic development and ultimately targeted treatments (Nadeau and Auwerx 2019).

In this Commentary, we discuss the study of the dark genome, the importance of pleiotropy and briefly the role of large-scale mouse functional genomics in analysing the dark genome and uncovering pleiotropy.

## The dark genome

Notwithstanding the extraordinary developments in determining the underlying loci and variants involved with disease, the programme for the Precision Medicine Initiative is severely hampered by the “dark” genome (Oprea et al. 2018; Oprea 2019). Surprisingly, we still know very little or nothing about the function of the majority of genes in the human and mouse genomes. The dark genome refers to those genes/proteins for which there is minimal knowledge on biological function and, allied to this, limited tools for their analysis (such as antibodies). Oprea et al. classify loci into four types—Tdark, Tbio, Tchem and Tclin. Tbio includes loci that have a confirmed Mendelian Disease phenotype; GO terms based on experimental evidence and two of the following three conditions: > 5 PubMed publication count; > 3 NCBI Gene Reference into Function (RIF) annotations; and > 50 commercial antibodies. Tbio loci represent genes for which there is a solid foundation for further deep drilling research, and they clearly lie outside of the dark genome. The Tchem and Tclin categories describe genes with even more advanced knowledgebases. Within the Tchem category are genes that encode proteins with known small molecules of high potency, while the Tclin category encompasses genes whose proteins are drug targets with at least one approved drug with known mechanism. Thus, Tdark lies at the bottom, in the murky underworld of molecular genetic research activity, with few if any PubMed publications, a low Gene RIF count, and limited antibodies. Moreover, a relatively small proportion of RO1 grants target Tdark and there are few patents which incorporate Tdark loci.

Most importantly, the dark genome is a barrier to the Precision Medicine Initiative. Firstly, the lack of knowledge on gene function compromises our ability to relate genetic variation to disease. As we discuss above, existing knowledge on gene and protein function is an important asset in interpreting and assigning causality to putative candidate variants. Secondly, the dark genome is bound to encompass many loci whose investigation would provide novel insights into genetic and physiological mechanisms, which would be transformative for our understanding of genome–phenotype relationships as well as identifying novel targets for therapeutic advances. Why does the dark genome persist and what can we do to illuminate this part of the genome?

## Why does the dark genome endure, and what is the remedy?

It is perplexing, particularly given the expanding resource and activity in functional genomics studies, why the dark genome persists. Oprea et al. (2018; Oprea 2019) suggest that the blame lies with a lack of tools to investigate the loci within the dark genome—a state which itself diminishes interest and the likelihood that useful tools will be generated. As a consequence, this vicious cycle endures and prevents these loci emerging from the dark.

Their thoughts resonate with another recent analysis by Stoeger et al. (2018) who have undertaken a comprehensive study of the reasons why genes in the dark genome are ignored. Genes that are studied in the past tend to be the ones that are studied in the future. They discovered that for the number of publications per gene there is a very significant correlation between ongoing current research output and that from preceding decades. Similarly, they find that there is a disproportionate attention in publications on already well-studied genes. Worryingly, they also uncover that previous knowledge on gene function along with the year of initial report are key factors in research funding, which is of course an important driver of future research activity. However, the authors also found that studies of human genes are significantly primed and enhanced by knowledge from model organisms—so examining publications on little-studied human genes and their homologues was more likely to have been encompassed within large-scale model organism functional studies. The corollary of this, confirmed by their studies, is that homologues of unstudied human genes have not been studied in model organisms. While on the face of it these observations may appear to be a setback for the delivery of research in novel hitherto unexplored regions of the genome, it underlines the importance and power of comprehensive model organism studies and the benefits of large-scale and agnostic genetic screens to uncover gene function across the entire genome landscape. These approaches represent a key route by which biomedical sciences can illuminate the dark genome and remove the roadblock on unstudied human genes, which impede the viability and progress of the Precision Medicine Initiative.

In summary, it will be necessary to develop and support research programmes that foster gene function studies across the entire genome and tackle those as yet unexplored loci that make up Tdark. Inevitably, phenotype and disease-driven studies in the human population will help uncover novelty within the dark genome, but it will be slow and incremental. Furthermore, the discovery of new loci in heterogeneous human populations is likely to miss discrete phenotypes and potentially thwart or limit our understanding of their functionality and the underlying mechanisms. Rather, the development of comprehensive large-scale functional screens in model organisms is a powerful and efficient way to ensure that functional knowledge spreads beyond the known genome and already well-studied loci. The evidence indicates that such programmes are a critical primer for novel studies of human gene function. These screens will need to assess, for each locus, the nature and extent of pleiotropy to provide rich and importantly causal insights into the diverse functions of individual genes and their complex and variable impact upon disease states.

## Pleiotropy

Pleiotropy is observed when a DNA variant determines multiple phenotypes. The phenomenon of pleiotropy has been recognised for 100 years or more (Stearns 2010) but has recently emerged as a seminal topic in human genetics given its importance in genomic and precision medicine. Pleiotropy manifests in disease as multi-morbidities. A critical question is the ubiquity of pleiotropy in human disease, which will be relevant for our understanding of disease mechanisms and the causal genetic pathways, as well as the relationship between genetic networks that underlie shared disease pathology. Critically, our knowledge of pleiotropy and how it impinges on the genetic underpinnings to disease will determine how effectively we can approach diagnosis, therapeutic intervention and individualised treatment within precision medicine.

Pleiotropy was recognisable for many years within plant and animal breeding programmes where it is common for selection on one particular trait to generate changes in other traits. However, a direct assessment of pleiotropy in mammals can be made by comprehensively phenotyping mouse mutants to define the number of traits that are associated with each gene (De Angelis et al. 2015; White et al. 2013). In these two studies, a significant number of loci exhibited multiple phenotypes. While a proportion of genes failed to exhibit a measurable phenotype and others had only one phenotype, it is unknown if the extension of phenotyping testing to additional systems and parameters would have revealed additional phenotype hits such that every gene might demonstrate pleiotropy. However, some quantitative assessments of the level of pleiotropy concluded that it was highly restricted in complex organisms (Wagner and Zhang 2011), but this has been contested (Hill and Zhang 2012a, b) and this view has not gained much ground.

Attention has turned to the examination of pleiotropy in the human population (Visscher and Yang 2016). Evidently, pleiotropy is present in humans as exemplified by a composite of phenotypes seen in disease syndromes caused by single-gene Mendelian disorders. It is noteworthy that the traits in syndromic disorders often encompass diverse biological systems. Importantly, analysis of the ever-expanding GWAS datasets is allowing us to identify loci that underlie multiple phenotypes and provide a robust view of the ubiquity of this phenomenon. It will be important to establish the causality of individual genes and genetic variants to definitively determine specific pleiotropic outcomes.

Pickrell et al. (2016) in their analysis of public domain GWAS data for 42 traits identified 341 loci that were pleiotropic, suggesting that pleiotropy was indeed common. Chesmore et al. (2018) have now extended this work and provided a comprehensive analysis of pleiotropy in the human genome, examining the entire GWAS catalogue of 1094 disease phenotypes and 14,459 genes. Pleiotropy was pervasive with the number of phenotypes per gene ranging from 1 to 53, and 44% of genes associated with more than one phenotype. Interestingly, it was found that the effect size scales with the degree of pleiotropy. One possible explanation is that genes with broad pleiotropic impacts are likely to represent critical physiological hubs and thus when mutated will exhibit stronger phenotypic effects. These physiological hubs have resonance with the model for complex traits proposed by Boyle et al. (2017), which envisages a modest number of core genes carrying large-effect variants that have a direct role in disease. However, their model also proposes that regulatory networks are so highly interconnected that potentially all genes will have effects on the core disease genes, though some of these effects may be vanishingly small. This “omnigenic” model would assume that pleiotropy is truly ubiquitous. However, the omnigenic model has recently been the subject of scrutiny from Visscher et al., who argue that the focus on key core disease genes may be misplaced, and that in terms of fully understanding polygenic architecture and overlying pleiotropy that study of the widest range of risk loci is required (Wray et al. 2018).

Gene variants may manifest pleiotropy through two different routes (sometimes referred to as Type 1 and Type 2 pleiotropy (Wagner and Zhang 2011). Firstly, a DNA variant may impact multiple traits directly through independent biological pathways, possibly due to tissue specific effects that reflect specific cellular functions or protein interactions. Secondly, a gene may affect a particular trait, through a single molecular function, whose perturbation secondarily impacts other traits. For the former, it is worth noting that different DNA variants within a gene might impact the relevant biological pathways to a greater or lesser extent, thus leading to heterogeneity between individuals in the degree and extent of pleiotropy observed. In addition, the landscape of core genes that affects a particular disease trait itself provides insights into the common genetic architecture that underlies the disease and is important for our knowledge of pleiotropy and multi-morbidities.

Overall, the intersection between the pleiotropy manifested via individual genes and the relationships between genes and pathways that underlie individual traits is the framework on which our understanding deepens of how perturbation of genetic networks causes disease. Moreover, this pleiotropic dissection of genetic networks will be critical for disease intervention and understanding how better to deliver treatment in a targeted and safe manner. To develop a more comprehensive knowledge of pleiotropy, we need not only to tackle the dark genome, but also to extend the reach of our functional studies within the known genome. Indeed, for many genes where causal relationships between genetic variant and disease traits have already been established, a complete knowledge of the pleiotropic spectrum and the full range of physiological impacts is unlikely to have been ascertained.

## Generating a comprehensive catalogue of network pleiotropy

The International Mouse Phenotyping Consortium (IMPC) is building a catalogue of mammalian gene functions by generating and phenotyping a knockout mouse line for every protein-coding gene (Brown and Moore 2012). To date, over 9000 knockout mouse lines, many for poorly understood genes, have been generated and over 6000 phenotyped in a coordinated effort involving 20 global research centres and dedicated publicly available online resources. Essentially, the IMPC phenotyping pipeline is designed to uncover pleiotropy systematically by investigating the mutational impact on multiple organ and disease systems. Testing platforms cover a wide range of behaviours, cardiac function, metabolism, fat deposition, vision, hearing, blood biochemistry, and bone morphology, among others, as well as post-mortem biochemistry and pathology with the aim to comprehensively catalogue all phenotypes determined by a gene. As we discuss above, in the pilot programmes which preceded IMPC such as the MGP programme at the Wellcome Trust Sanger Institute (White et al. 2013) and the EUMODIC programme (De Angelis et al. 2015) in which each studied several hundred genes, pleiotropy was found to be highly prevalent. The findings from IMPC, capturing a significantly greater proportion of the genome, are no different. It is startling that over 90% of all gene–phenotype relationships are novel and hitherto unreported (Meehan et al. 2017).

IMPC has now examined around one-third of the coding genome and is, for the first time, revealing the pleiotropic functions and multi-morbidities associated with many previously unannotated genes that are part of the dark genome. Moreover, it provides further insights into the pleiotropic nature of previously studied loci. Overall, it is playing a significant role in illuminating the dark genome, as well as enriching our knowledge of pleiotropy across all loci.

The large multidimensional datasets of IMPC (> 75 M datapoints) enable an unprecedented view of the mammalian genome landscape, particularly in revealing novel loci, many of them thus far unstudied, associated with various disease states (Meehan et al. 2017). Studies of homozygous embryonic lethal mutations have reinforced the enrichment of human Mendelian disease genes among this class of loci (Dickinson et al. 2016). Both female and male mutant mice enter the IMPC pipeline and this has enabled a better understanding of the pervasive and wide-ranging sexual dimorphism of phenotypic traits in both wild-type and mutant mice (Karp et al. 2017). Importantly, the identification of novel disease genes and mechanisms in areas as diverse as metabolism (Rozman et al. 2018), hearing (Bowl et al. 2017) and vision (Moore et al. 2018) have uncovered an unexplored genetic landscape of disease. The generation of an unbiased dataset linking all genes to comprehensive phenotype information will allow us to begin to explore the nature of pleiotropy on a genome-wide scale. Moreover, the disease models generated by IMPC along with the associated phenotype data allow us to dissect further the nature and interplay across multiple loci and genetic pathways of multi-morbidity in disease states.

## Conclusion

The plethora of new genetic disease models generated by IMPC and others, together with knowledge on basic gene function and pleiotropy, will inform and underpin studies on rare diseases and Mendelian disorders, illuminate GWAS studies and ultimately help provide a more profound understanding of the function of human genetic variation and its involvement in disease. The study of the dark genome alongside the generation of a comprehensive map of the pleiotropic functions of all genes is critical to informing a deeper understanding of the mammalian genome. Future advances in genomic and precision medicine will depend upon the success of this endeavour.

## References

[CR1] Bowl MR, Simon MM, Ingham NJ (2017). A large scale hearing loss screen reveals an extensive unexplored genetic landscape for auditory dysfunction. Nat Commun.

[CR2] Boyle EA, Li YI, Pritchard JK (2017). An expanded view of complex traits: from polygenic to omnigenic. Cell.

[CR3] Brown SDM, Moore MW (2012). The international mouse phenotyping consortium: past and future perspectives on mouse phenotyping. Mamm Genome.

[CR4] Chesmore K, Bartlett J, Williams SM (2018). The ubiquity of pleiotropy in human disease. Hum Genet.

[CR5] Collins FS, Varmus H (2015). A new initiative on precision medicine. N Engl J Med.

[CR6] De Angelis MH, Nicholson G, Selloum M (2015). Analysis of mammalian gene function through broad-based phenotypic screens across a Consortium of Mouse Clinics. Nat Genet.

[CR7] Dickinson ME, Flenniken AM, Ji X (2016). High-throughput discovery of novel developmental phenotypes. Nature.

[CR8] Gurdasani D, Barroso I, Zeggini E, Sandhu MS (2019). Genomics of disease risk in globally diverse populations. Nat Rev Genet.

[CR9] Hill WG, Zhang X-S (2012). On the pleiotropic structure of the genotype-phenotype map and the evolvability of complex organisms. Genetics.

[CR10] Hill WG, Zhang X-S (2012). Assessing pleiotropy and its evolutionary consequences: pleiotropy is not necessarily limited, nor need it hinder the evolution of complexity. Nat Rev Genet.

[CR11] Karp NA, Mason J, Beaudet AL (2017). Prevalence of sexual dimorphism in mammalian phenotypic traits. Nat Commun.

[CR12] Meehan TF, Conte N, West DB (2017). Disease model discovery from 3,328 gene knockouts by The International Mouse Phenotyping Consortium. Nat Genet.

[CR13] Moore BA, Leonard BC, Sebbag L (2018). Identification of genes required for eye development by high-throughput screening of mouse knockouts. Commun Biol.

[CR14] Nadeau JH, Auwerx J (2019). The virtuous cycle of human genetics and mouse models in drug discovery. Nat Rev Drug Discov.

[CR15] Oprea T (2019). Exploring the dark genome: implications for precision medicine. Mamm Genome.

[CR16] Oprea TI, Bologa CG, Brunak S (2018). Unexplored therapeutic opportunities in the human genome. Nat Rev Drug Discov.

[CR17] Pickrell JK, Berisa T, Liu JZ (2016). Detection and interpretation of shared genetic influences on 42 human traits. Nat Genet.

[CR18] Riordan JD, Nadeau JH (2017). From peas to disease: modifier genes, network resilience, and the genetics of health. Am J Hum Genet.

[CR19] Rozman J, Rathkolb B, Oestereicher MA (2018). Identification of genetic elements in metabolism by high-throughput mouse phenotyping. Nat Commun.

[CR20] Stearns FW (2010). One hundred years of pleiotropy: a retrospective. Genetics.

[CR21] Stoeger T, Gerlach M, Morimoto RI, Nunes Amaral LA (2018). Large-scale investigation of the reasons why potentially important genes are ignored. PLoS Biol.

[CR22] Visscher PM, Yang J (2016). A plethora of pleiotropy across complex traits. Nat Genet.

[CR23] Wagner GP, Zhang J (2011). The pleiotropic structure of the genotype-phenotype map: the evolvability of complex organisms. Nat Rev Genet.

[CR24] White JK, Gerdin A-K, Karp NA (2013). Genome-wide generation and systematic phenotyping of knockout mice reveals new roles for many genes. Cell.

[CR25] Wray NR, Wijmenga C, Sullivan PF (2018). Common disease is more complex than implied by the core gene omnigenic model. Cell.

